# The mycolic acid reductase Rv2509 has distinct structural motifs and is essential for growth in slow‐growing mycobacteria

**DOI:** 10.1111/mmi.14437

**Published:** 2019-12-17

**Authors:** Asma Javid, Charlotte Cooper, Albel Singh, Steffen Schindler, Milena Hänisch, Robert L. Marshall, Rainer Kalscheuer, Vassiliy N. Bavro, Apoorva Bhatt

**Affiliations:** ^1^ School of Biosciences and Institute of Microbiology and Infection University of Birmingham Birmingham UK; ^2^ Institute of Pharmaceutical Biology and Biotechnology Heinrich Heine University Düsseldorf Düsseldorf Germany; ^3^ School of Life Sciences University of Essex Colchester UK

**Keywords:** dehydrogenase, *Mycobacterium*, mycolic acid, reductase, tuberculosis

## Abstract

The final step in mycolic acid biosynthesis in *Mycobacterium tuberculosis* is catalysed by mycolyl reductase encoded by the *Rv2509* gene. Sequence analysis and homology modelling indicate that Rv2509 belongs to the short‐chain fatty acid dehydrogenase/reductase (SDR) family, but with some distinct features that warrant its classification as belonging to a novel family of short‐chain dehydrogenases. In particular, the predicted structure revealed a unique α‐helical C‐terminal region which we demonstrated to be essential for Rv2509 function, though this region did not seem to play any role in protein stabilisation or oligomerisation. We also show that unlike the *M. smegmatis* homologue which was not essential for growth, *Rv2509* was an essential gene in slow‐growing mycobacteria. A knockdown strain of the *BCG2529* gene, the *Rv2509* homologue in *Mycobacterium bovis* BCG, was unable to grow following the conditional depletion of BCG2529. This conditional depletion also led to a reduction of mature mycolic acid production and accumulation of intermediates derived from 3‐oxo‐mycolate precursors. Our studies demonstrate novel features of the mycolyl reductase *Rv2509* and outline its role in mycobacterial growth, highlighting its potential as a new target for therapies.

## Graphical abstract

The *Mycobacterium tuberculosis* gene *Rv2509* encodes a reductase enzyme involved in the last step of synthesis of mycolic acids, a key cell wall lipid. By generating a predicted model for the protein encoded by *Rv2509*, we show that the enzyme has distinct structural motifs including a distinct C‐terminal region, which we show by complementation studies to be vital for function. The gene is also essential for growth and viability underlining its potential as a new drug target.

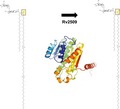

## 1 INTRODUCTION

**Figure 1:**
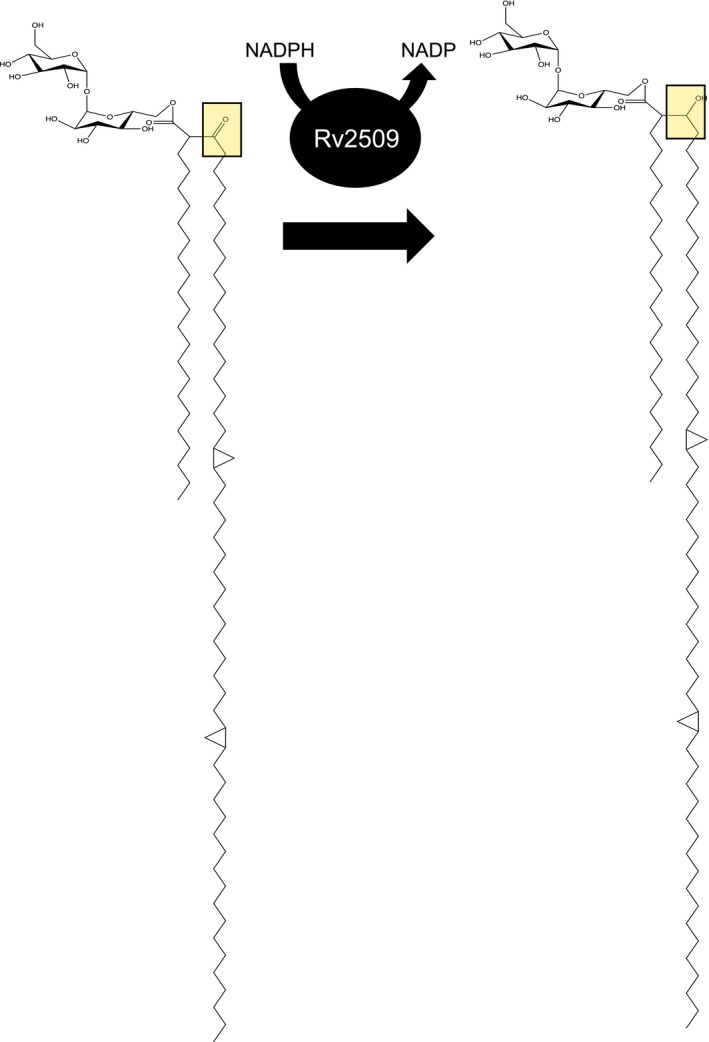
Proposed reaction catalysed by Rv2509. The putative substrate is trehalose mono‐premycolate and the product is trehalose monomycolate. The examples shown here is that of a α‐mycolic acid from *Mycobacterium tuberculosis* [Colour figure can be viewed at https://www.wileyonlinelibrary.com]

The cell walls of mycobacteria including the tuberculosis‐causing *Mycobacterium tuberculosis* contain distinct long‐chain fatty acids termed mycolic acids. These α‐alkyl, β‐hydroxy fatty acids form an integral part of the cell wall of mycobacteria, and of other related bacteria of the suborder Corynebacterineae, including the genera *Nocardia*, *Corynebacterium* and *Rhodococcus* (Marrakchi, Laneelle, & Daffe, 2014). Mycolic acids can be found covalently linked to cell wall arabinogalactan and as part of the glycolipids' trehalose monomycolate (TMM), trehalose dimycolate and glucose monomycolate. They are essential for mycobacterial viability (Marrakchi et al., 2014; Nataraj et al., 2015) and virulence and are synthesised by a complex array of enzymes that includes a mammalian‐like Fatty Acid Synthase I (FAS‐I), and a multienzyme complex of Fatty Acid Synthase‐II (FAS‐II) (Marrakchi et al., 2014). One of the late stages of mycolic acid biosynthesis involves the Claisen condensation of a FAS‐II‐derived long meromycolate chain (C_42_‐C_62_) with a short FAS‐I‐derived fatty acid (C_24_‐C_26_) to yield α‐alkyl, β‐keto fatty acid intermediate, catalysed by the polyketide synthase Pks13 (Gande et al., 2004; Portevin et al., 2004). The final step in the formation of a mature mycolic acid involves the enzymatic reduction of the β‐keto group of the product of Pks13 to produce α‐alkyl, β‐hydroxy fatty acids (mature mycolic acid). In *M. tuberculosis*, the reductase required for this final step is encoded by *Rv2509* (Bhatt, Brown, Singh, Minnikin, & Besra, 2008; Lea‐Smith et al., 2007). Initial studies in *Corynebacterium glutamicum*, a species that can survive the loss of mycolic acids, indicated that deletion of *cmrA* (*NCgl2385*), the *Rv2509* orthologue, resulted in a strain that accumulated the α‐alkyl, β‐keto fatty acid precursor instead of mature mycolic acids (Lea‐Smith et al., 2007). Mycolic acid biosynthesis genes are essential in mycobacteria; however, surprisingly, we were able to generate a viable mutant of *MSMEG4722*, the homologue of *Rv2509* in the fast‐growing *Mycobacterium smegmatis* (Bhatt et al., 2008). Similar to the *C. glutamicum NCgl2385* mutant, the *M. smegmatis MSMEG4722* mutant produced α‐alkyl, β‐keto fatty acyl precursors of mycolic acids (3‐oxo‐mycolic acid precursors) which were transported and esterified to arabinogalactan in the cell wall (Bhatt et al., 2008). While the precise knowledge of the substrate for *Rv2509* is lacking, functional analysis of the preceding enzyme in the biosynthetic pathway, Pks13, indicated that, while this polyketide synthase primarily catalyses the formation of the α‐alkyl, β‐keto fatty acyl precursor, it also contains enzymatic motifs that facilitate the release of the nascent fatty acyl chain and its subsequent transfer to a trehalose residue to produce a trehalose residue esterified with the α‐alkyl, β‐keto fatty acyl precursor of mycolic acid (Gavalda et al., 2014). These findings suggest that the trehalose‐bound mono 3‐oxo‐mycolic acid precursor may likely be the substrate for Rv2509, which catalyses its conversion to TMM (Figure 1). In this study, we set out to do a detailed study of the amino acid sequence of Rv2509 with the aim of deducing its structure and functional connections, which led to the identification of series of unique sequences, and by proxy, novel structural features. We then probed the role of one distinct structural feature of the mycobacterial mycolyl reductase in vitro by deletion analysis. We also probed the essentiality of *Rv2509* for the growth and viability of the slow‐growing *M. tuberculosis* complex using *Mycobacterium bovis* BCG.

## 2 RESULTS

### 2.1 Homologues of *M. tuberculosis Rv2509* are found across mycolic acid‐producing species

**Figure 2:**
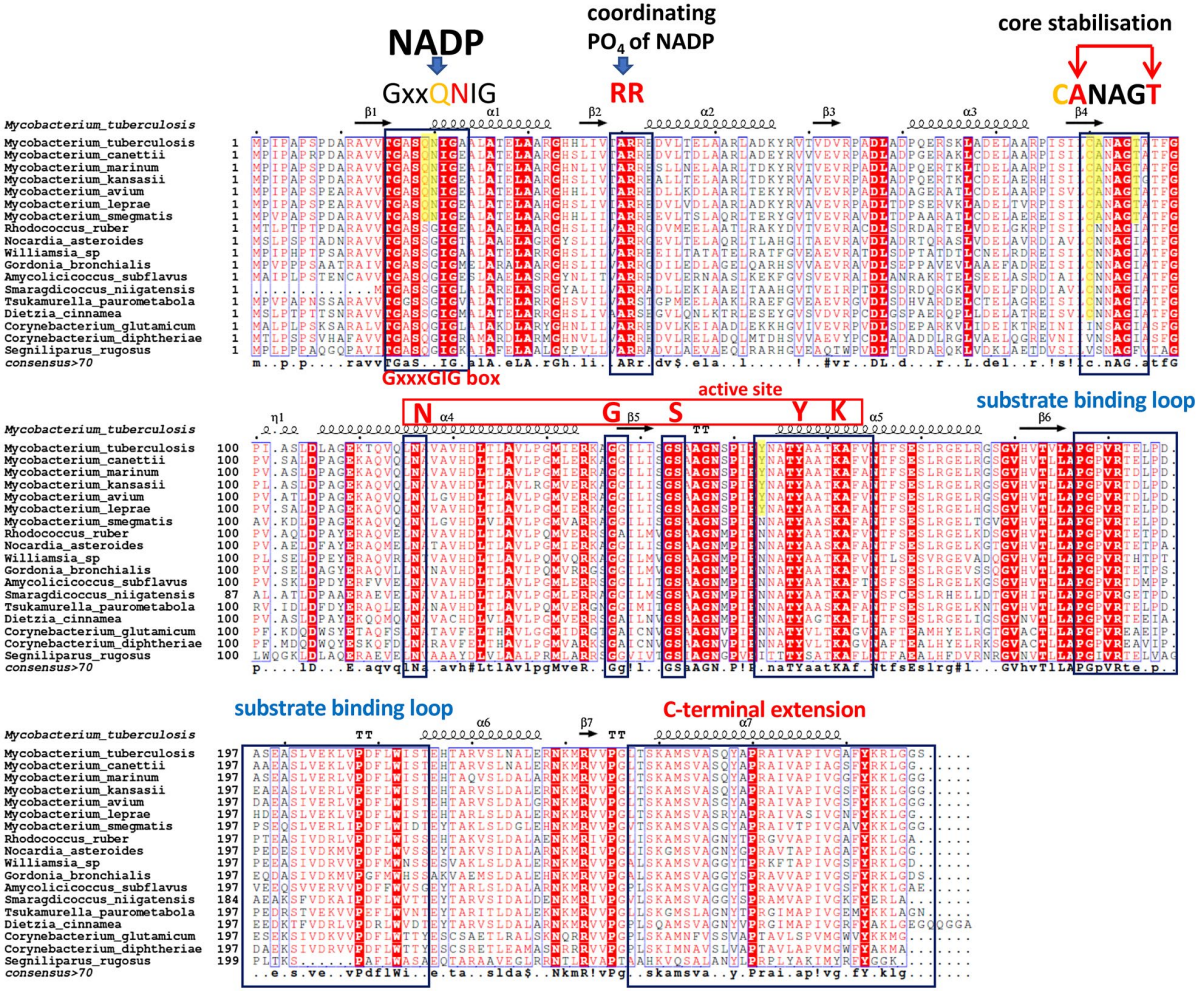
A structural alignment of the predicted Rv2509 structure from *Mycobacterium tuberculosis,* with annotated secondary structure elements (top) with the sequences of the Mycobacterial and Corynebacterial homologues highlighting the unique sequence motifs of the *Rv2509* subfamily of SDRs. The GxxQNIG motif along with RR pair identifies a novel NADP(H) binding consensus, limited to *Mycobacteriaceae* (highlighted in yellow). GxxSGIG is the wider consensus seen in the more remotely related Corynebacteria. Similarly, the unique Rossmann core‐stabilisation signature CANAGT reported here is also restricted to the *Mycobacteriaceae* (highlighted in yellow) and reverts to the more common (C)NNAG(I/F) in the more remote homologues. Active site residues N‐G‐S‐Y‐K in alpha‐helix 4 and alpha‐helix 5 are preserved throughout the family; however, a unique additional tyrosine residue can be seen to be present in *Mycobacteriaceae* (highlighted in yellow)*.* The C‐terminal extension is also uniquely conserved across the wider Corynebacterial species

**Figure 3:**
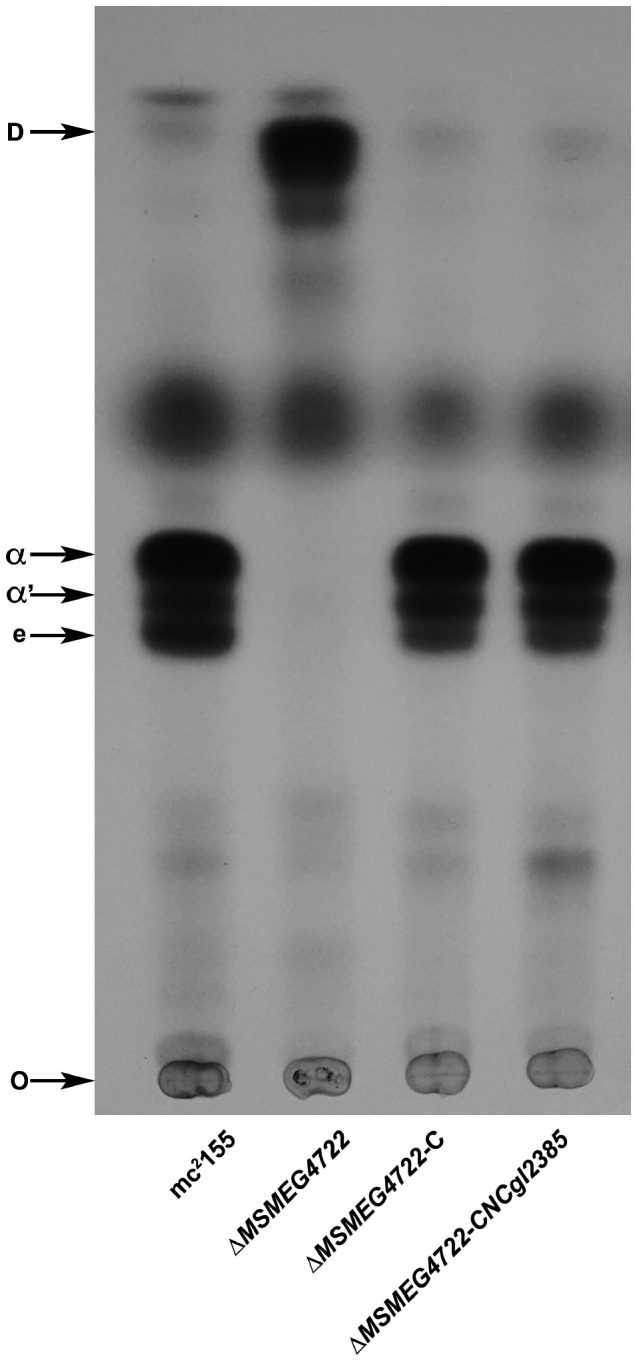
Complementation of the *M. smegmatis* Δ*MSMEG4722* mutant with the Corynebacterial mycolyl reductase. TLC analysis of ^14^C‐labelled mycolic acid methyl esters (MAMEs) isolated from different strains. α; α MAMEs, α′; α′ MAMEs, e; epoxy MAMEs, O; origin, D, degradation products derived from 3‐oxo‐mycolate precursors. Solvent system; petroleum ether:acetone (95:5, v:v)

While mycolic acids are produced by several species belonging to the suborder Corynebacterineae, the biosynthesis of mycolates has been studied in detail exclusively in the genera *Mycobacterium* and *Corynebacterium*. While the two genera share a common set of enzymes required for mycolic acid biosynthesis, select differences also exist. For example, while the meromycolate chains are synthesised by a Type‐II fatty acid synthase complex in species of *Mycobacterium*, the same function is carried out by a mammalian‐like Type‐I fatty acid synthase in *Corynebacterium* species (Radmacher et al., 2005). Similarly, differences exist in the transport of mycolic acids between the two genera (Varela et al., 2012; Yang et al., 2014). To investigate whether the reduction of premycolates by an exclusive mycolyl reductase, was an enzymatic process conserved across other mycolic acid‐producing species, we used the amino acid sequence of Rv2509 as a source template for a BLASTp search of genome sequences of other known mycolic acid‐producing bacteria. Homologues of *Rv2509* were found across the mycolate‐producing genera (Figure 2). A phylogenetic analysis of *Rv2509*, its homologues in other mycobacteria and in select mycolic acid‐producing species revealed that mycolyl reductases from Corynebacterial species branched early (Supporting information Figure S1). Corynebacteria produce relatively short chain mycolic acids (Radmacher et al., 2005). To test whether mycolyl reductases across the mycolata had evolved specificities to accommodate differing chain lengths, we transformed the *M. smegmatis* Δ*MSMEG4722* strain with a plasmid‐containing *C. glutamicum* mycolyl reductase gene *NCgl2385* to generate the strain Δ*MSMEG4722*‐C*NCgl2385*. Mature mycolic acid production was restored in the transformed strain, indicating that *NCgl2385* could reduce *M. smegmatis* 3‐oxo‐mycolic acid precursors and consequently fully complement the mutant *M. smegmatis* strain. This outcome suggested that mycolyl reductases across the mycolata likely did not evolve specificities for differing chain lengths (Figure 3).

### 2.2 Predicted structure of *M. tuberculosis* Rv2509

We attempted to deduce possible functional properties of Rv2509 using a comparative sequence and structural analysis with proteins of known function. Sequence analysis of Rv2509 using the NCBI conserved domain database (Marchler‐Bauer et al., 2015) categorises the protein as a part of the DltE family of short‐chain dehydrogenases (cluster COG0300), which are in turn members of the cl27753 short‐chain dehydrogenase/reductase (SDR) superfamily of NAD(P)‐dependent oxidoreductases. This is a diverse group, containing over 80,000 unique sequences (Fujisawa, Nagata, & Misono, 2003). However, despite this diversity, all SDR‐superfamilies display the classical Rossmann fold typical of NAD^+^/NADP^+^‐binding proteins and hence can be classified as belonging to the cl21454 family of topologically and structurally related proteins.

**Figure 4:**
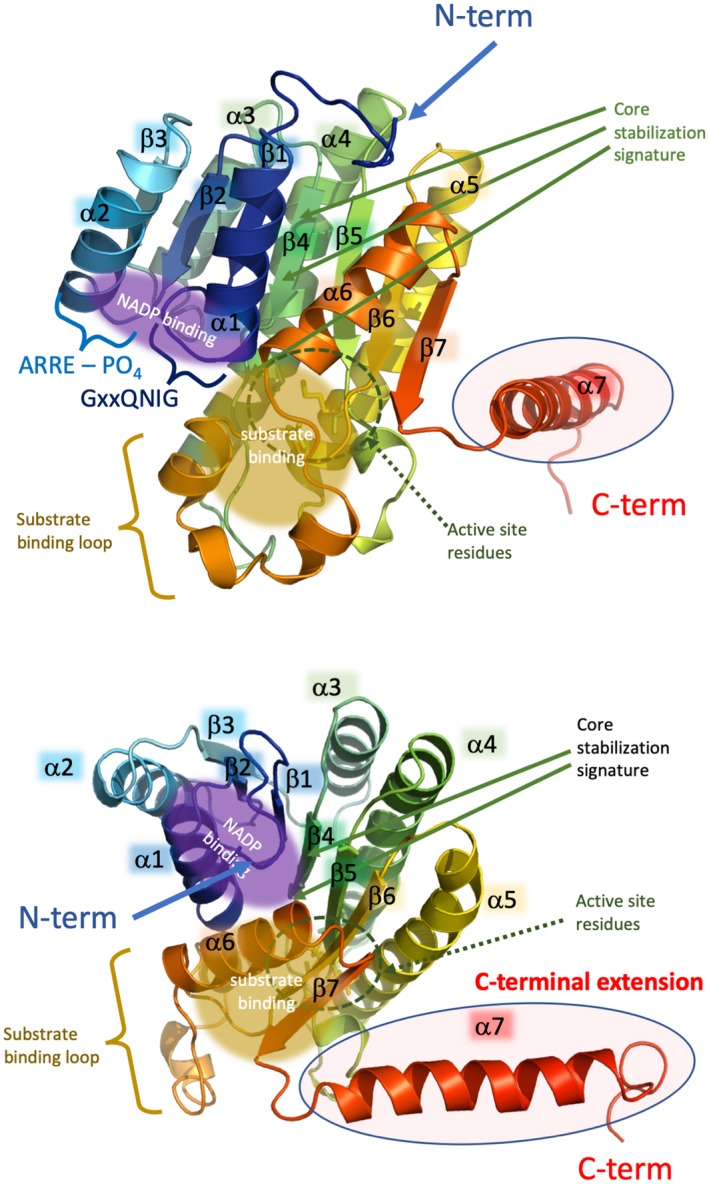
Visualisation of the predicted structure of *Mycobacterium tuberculosis* Rv2509 monomer. Two different orientations are given; side‐view and top‐down view towards the coenzyme binding site. The protein is coloured like a rainbow from blue (N‐terminus) to red (C‐terminus) and the main secondary structure elements are labelled, as well as the predicted C‐terminal extension domain is circled in a red oval. The location of the key sequence and structural motifs discussed in the text is also provided

Next, we screened the peptide sequence of *Rv2509* against the PDB database to find structural templates for homology modelling. The serine dehydrogenase YdfG (UniProt ID P39831; PDB ID 3ASU/3ASV) (Yamazawa, Nakajima, Mushiake, Yoshimoto, & Ito, 2011) had the highest identity score; however, structural analysis revealed several gaps in the pairwise alignment and the C‐terminal region was shorter than that observed in *Rv2509,* which necessitated additional template input. Therefore, the structures of the four highest‐scoring experimental SDRs (3ASU/3ASV, 1XG5, 4X54 and 4BMV) were used in parallel as a basis for comparative homology modelling of *Rv2509* (Figure 4) using the I‐TASSER package (Yang et al., 2015) with specific template selection enabled. Notably, while, three of the templates yielded models with roughly equivalent (confidence) C‐scores, the one based on the SDR from *Sphingobium yanoikuyae* (4BMV.pdb; GenBank ID ACB78183.1) stood out with the highest C‐score of 0.6, indicating a high quality of prediction.

As visualised by the structural alignment (Figure 2) which shows the predicted secondary structure elements of Rv2509 aligned to its Corynebacterial homologues, the core structure of the Rv2509 model presents a typical NADP‐binding protein Rossmann fold (residues 1–235) (Persson, Kallberg, Oppermann, & Jornvall, 2003) with seven parallel β‐strands forming a central β‐sheet, which is sandwiched between two layers of α‐helices—three on each side (Figure 4). Although an earlier study has correctly classified Rv2509 within the short‐chain reductase (SDR) superfamily and identified crucial sequence signatures such as the potential NAD(H)/NADP(H)‐binding motif and active site in residues 157–161 (Lea‐Smith et al., 2007), it did not include further structural prediction and analysis, and was limited in terms of sequences used. We, therefore, sought to expand this initial analysis by performing a systematic analysis of the Rv2509 orthologues from a wide range of *Corynebacterineae* to represent all known mycolate‐producing genera.

We initiated these studies by analysing the cofactor binding site. SDRs can be divided into two large families, “Classical” SDRs which are around 250 amino acid residues long and “Extended” SDRs which contain around 350 residues (Jornvall et al., 1995), exhibiting differences in the arrangement of glycine residues within the coenzyme binding sites. Coenzyme preference‐predictions based on the strong conservation of the signature residues (Persson et al., 2003) classified the Rv2509 into the group of the NADP(H)‐binding SDRs, consistent with its mycolyl reductase function. However, it was difficult to categorically allocate Rv2509 into a specific group of SDRs as the predicted NADP‐binding site of *Rv2509* (G_16_XXQ_19_N_20_I_21_G_22_ **)** displays a unique and novel variation of the (T)‐G‐X‐X‐X‐G‐I‐G motif described by Brakoulias and Jackson (2004) (Figure 2). The variation seems to be restricted to the long‐chain mycolates‐producing species of *Mycobacteriaceae*, as short‐chain mycolates‐producing Corynebacteria retain the more established G‐X‐X‐X‐G‐I‐G motif. (Figure 2). Key residue positions in the NADP(H) site suggest that *Rv2509* is a cP2 member of the “Classic” SDR family, according to the classification system of Persson et al., (Brakoulias & Jackson, 2004), which is further reinforced by the presence of two specific discriminator residues, R‐R, found in positions 41–42 in both *Rv2509* and YdfG from *Escherichia coli* (3ASV.pdb), as these residues are associated with coordination of the phosphate group of the NADP(H) cofactor. Further analysis of *Rv2509* suggests that it has a Class IV NADP(H)‐binding site (with consensus [AVIC]‐[LIV]‐[VIL]‐T‐G‐[ASGC]‐X_2_‐[GR]‐[ILF]‐G‐X_6_‐[LFY] (Hua, Wu, Sargsyan, & Lim, 2014). Thus, our analysis firmly places the Rv2509 family within the NADP(H)‐binding subfamily of SDRs.

The active site in SDRs is formed by a tight cleft, lined by α‐helix 4, β‐strand 4 and α‐helix 5 (Filling et al., 2002; Oppermann et al., 1997). Rv2509 exhibits the “classical” SDR motif Yx[AS][ST]K in α‐helix 5 (Y157 and K161 being the central catalytic residues), while the GxggxgSS/T motif in β‐strand 4 (G137, S144) and the central residue of α‐helix 4, N116 (Figure 2) are also instantly recognisable. In addition, it is possible that some contact with the substrate may be provided by the β‐strand 4.

Rather intriguingly, the consensus residues “NNAG” involved in the stabilisation of the central β‐sheet of the Rossmann fold in the “Classical” SDRs and located at the core of the protein are not fully conserved in *Rv2509* but take the form of a novel “ANAG” sequence (see Figure 2). Furthermore, this novel sequence is paired with a preceding Cys residue, which is also correlated with the presence of a C‐terminal Thr (in position 91 in *M. tuberculosis*). This unique “CANAGT” signature makes *Rv2509* orthologues in *Mycobacteriaceae* instantly recognisable (Figure 2).

In SDR proteins, the substrate‐binding loop links the sixth β‐sheet with the seventh α‐helix and is one of the larger loops. In the predicted structure of Rv2509 this loop spans roughly from P_187_ to S_214_ and appears largely conserved within the mycolic acid‐producing species (Figure 2). While the loop's length and position corresponds closely to the length of the substrate‐binding loop in both 4BMV (P183‐E206) and 3ASV (P178‐T208), there was little conservation of the loop's sequence relative to Rv2509 in these and other divergent SDRs (see ConSurf analysis below (Ashkenazy et al., 2016); Supporting information Figure S2), suggesting, as expected, major differences in the structure of the substrate for Rv2509.

The most intriguing finding upon the analysis of the *Rv2509* orthologues was that all of them seem to present a C‐terminal domain which seems to be highly specific to the *Corynebacterineae*. It proved also the most difficult to model reliably, as in the majority of the available SDR structural templates, the C‐terminal region, appears to be either truncated or disorganized. ConSurf mapping using the multiple sequence alignment shown in Figure 2 and multiple sequence alignment based on 150 nonredundant sequences of remote SDR homologues revealed that the C‐terminus of Rv2509 is uniquely a feature of the subfamily of Corynebacteriales (Supporting information Figure S3). Both pairwise and multiple sequence analyses indicated that the C‐terminal region of the 4BMV.pdb is closely related to Rv2509 and thus can be tentatively used as a template for that region. This was further corroborated that by comparative modelling (Song et al., 2013) and pure *ab‐initio* modelling (Raman et al., 2009), as implemented in the NewRobetta server (http://new.robetta.org). To validate this hypothesis, we conducted additional ab initio secondary structure predictions of *Rv2509* (236–268) using JPred4 (Drozdetskiy, Cole, Procter, & Barton, 2015) and PSIPRED (Buchan & Jones, 2019; Jones, 1999). Both of these bioinformatics tools supported a high alpha‐helical propensity of the C‐terminal region of *Rv2509*, suggesting that 4BMV as a reliable template for the region (Supporting information Figure S3). Indeed, the C‐terminus of the 4BMV.pdb structure, similarly to *Rv2509,* appears to be extended and is well‐structured, presenting an α‐helical organisation.

### 2.3 The extended C‐terminal of Rv2509 is essential for the function

**Figure 5:**
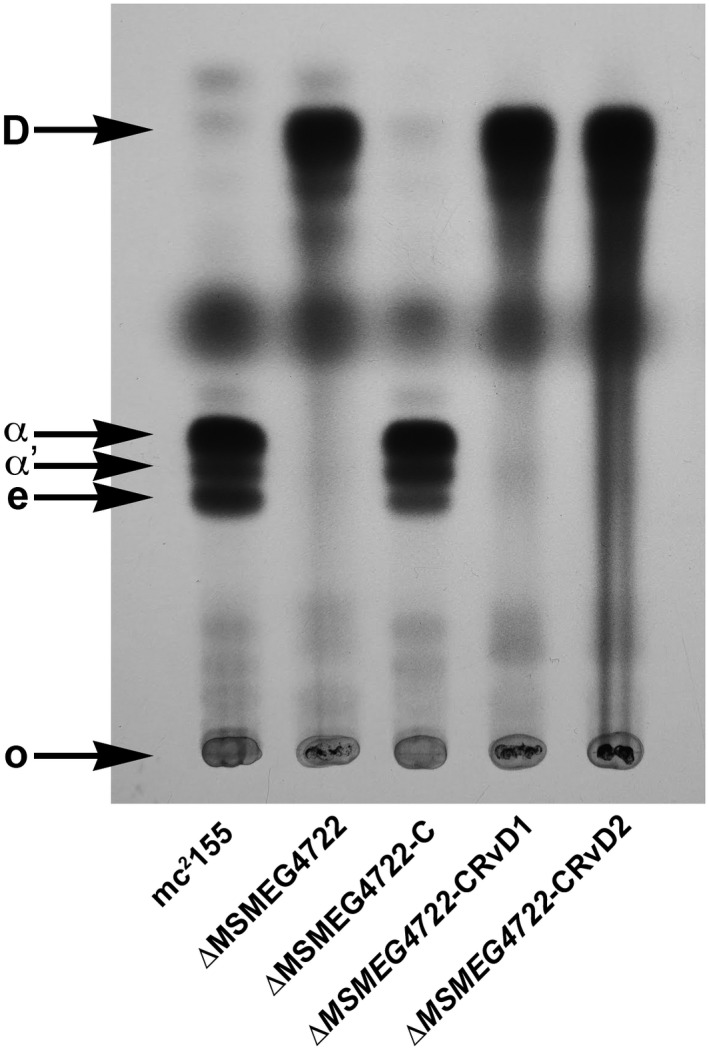
TLC analysis of ^14^C‐labelled MAMEs isolated from the strains of *M. smegmatis* Δ*MSMEG4722* mutant transformed with the different deletion constructs of *Rv2509*. α; α MAMEs, α′; α′ MAMEs, e; epoxy MAMEs, O; origin, D, degradation products derived from 3‐oxo‐mycolate precursors. Solvent system; petroleum ether:acetone (95:5, v:v)

Mature mycolic acid production could be restored in the *M. smegmatis* Δ*MSMEG4722* mutant on complementation with the *M. tuberculosis* orthologue *Rv2509* (Bhatt et al., 2008). The mutant strain thus provided us with a means of identifying residues and domains required for Rv2509 function, by assessing the ability of mutated constructs of plasmid‐borne *Rv2509* to complement the Δ*MSMEG4722* strain by restoring mature mycolic acid biosynthesis. To test if the extended C‐terminal of Rv2509 was required for function, we generated truncated versions of *Rv2509* cloned in the replicative shuttle plasmid pMV261 (Stover et al., 1991). Individual plasmids were then introduced by electroporation into the *M. smegmatis* Δ*MSMEG4722* mutant and the transformed strain analysed for the restoration of mature mycolic acid biosynthesis. Deletion of 14 amino acid residues (Ala_255_‐Ser_268_) from the C‐terminal of Rv2509 resulted in a complete loss of function as the strains transformed with the truncated construct failed to restore mature mycolic acid biosynthesis in the Δ*MSMEG4722* strain (Figure 5; Δ*MSMEG4722‐*CRvD1). A construct with a shorter truncation (Tyr_262_‐Ser_268_ deleted) also failed to complement the Δ*MSMEG4722* strain (Figure 5; Δ*MSMEG4722‐*CRvD2). Expression of *Rv2509* and truncated constructs could be detected by RT‐PCR confirming that the loss of Rv2509 function was linked to the deletion of the C‐terminal residues (Supporting information Figure S4). These results demonstrated that the C‐terminus of Rv2509 spanning terminal residues Ala_255_‐Ser_268_ was critical for function. The findings suggested an essential role for the unique, C‐terminal extension in the activity of Rv2509.

To further query whether this C‐terminal extension played a role in either protein stability, protecting Rv2509 from degradation, or in oligomerisation, we expressed N‐terminal His‐tagged versions of Rv2509 and Rv2509‐D1 (Rv2509 with Ala_255_‐Ser_268_ deleted) in *E. coli*. Western blot analysis of native polyacrylamide gels showed that there were no patterns of degradation detected in Rv2509‐D1, indicating that the loss of function due to the deletion of this region was not related to protein stability (Supporting information Figure S5). Furthermore, we did not observe additional slower migrating bands, indicating dimerisation (or oligomerisation) of full‐length or truncated Rv2509. Furthermore, cross‐linking Rv2509 with glutaraldehyde did not reveal additional slower migrating bands, suggesting that Rv2509 did not oligomerise in solution (Supporting information Figure S5).

### 2.4 *Rv2509* is an essential mycobacterial gene

Given the nonessentiality of the mycolyl reductase gene in *M. smegmatis* and prediction of transposon‐site hybridisation screens of a slow‐growing *M. tuberculosis Rv2509* mutant, we aimed to generate a null mutant of *Rv2509* in *M. tuberculosis* using Specialised Transduction (Bardarov et al., 2002). Such a mutant would also allow us to test the role of the mycolyl reductase in virulence using macrophage and mouse models of infection. However, repeated attempts failed to generate transductants, suggesting that *Rv2509* was essential for growth in *M. tuberculosis*. Indeed, subsequent transposon mutagenesis screens utilising deep sequencing predicted *Rv2509* to be essential for in vitro growth of *M. tuberculosis* (Griffin et al., 2011). We then utilised a promoter replacement strategy to demonstrate the essentiality of *Rv2509* in slow‐growing mycobacterial vaccine strain *Mycobacterium bovis* BCG. The P*myc1* promoter from *M. smegmatis* engineered to contain four *tetO* operator sites (Korte et al., 2016) was inserted immediately upstream of the start codon of *BCG2529,* the *Rv2509* homologue in *M. bovis* BCG‐Pasteur, using Specialised Transduction generating the strain BCG::P_Tet_‐*BCG2529*. Controlled gene expression of the *BCG2529* gene was achieved using a plasmid‐borne synthetic gene (*rev‐tetR*) derived from Tn10 *tetR* encoding a mutated TetR protein with reversed binding affinity to *tetO* sites upon the binding of tetracycline. The addition of anhydrotetracycline (ATc) results in the loss of expression.

**Figure 6:**
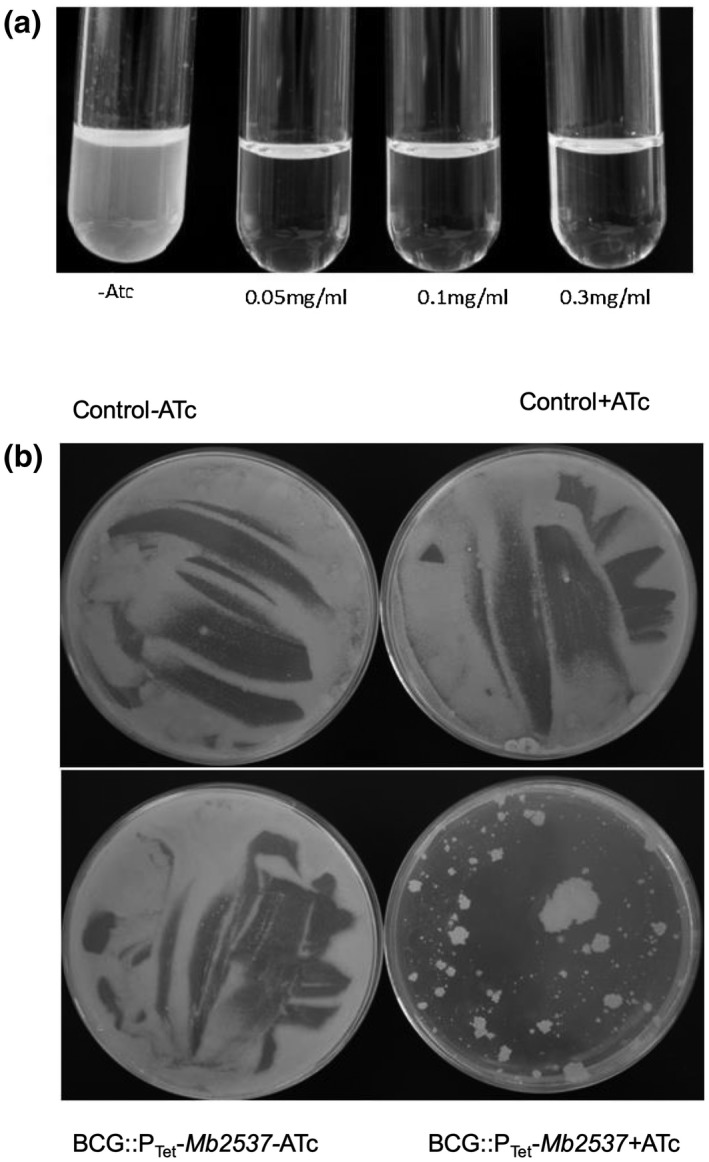
Conditional depletion of the mycolyl reductase in BCG::P_Tet_‐*BCG2529* leads to the loss of growth. (a) growth in different concentrations of ATc in 7H9 broth, (b) growth on 7H10 agar containing 5 µg/ml of ATc. Control strain; BCG transformed with pMV261::*rev‐tetR*‐RBS‐D

The addition of ATc resulted in the loss of growth of BCG::P_Tet_‐*BCG2529* in broth, indicating that the expression of the gene encoding the mycolyl reductase was essential for the in vitro growth of slow‐growing mycobacteria (Figure 6a). Furthermore, while concentrated cultures of BCG::P_Tet_‐*BCG2529* showed confluent growth on 7H10 agar plates, no confluent growth was observed on plates containing ATc (Figure 6b). We did observe a few scattered colonies on the ATc‐containing plates and these are likely to be suppressors allowing leaky expression of P_Tet_ driven *BCG2529*. These results demonstrated that the mycolyl reductase was essential for the growth and viability of slow‐growing mycobacteria.

### 2.5 Conditional depletion of the mycolyl reductase results in the loss of mature mycolic acids

**Figure 7:**
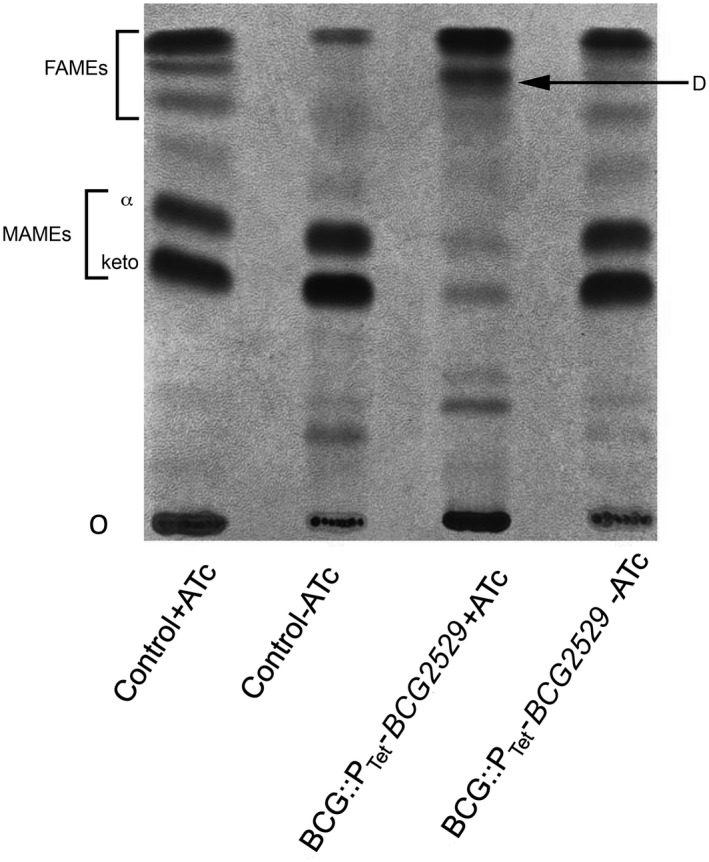
TLC analysis of ^14^C‐labelled MAMEs isolated from BCG::P_Tet_‐*BCG2529* strain. α; α MAMEs, keto; keto MAMEs, O; origin, D, degradation products derived from 3‐oxo‐mycolate precursors. Solvent system; petroleum ether:acetone (95:5, v:v)

The *M. smegmatis* mycolyl reductase mutant was unable to synthesise fully mature mycolic acids and instead produced 3‐oxo‐mycolate precursors that were found esterified to the cell wall arabinogalactan and to trehalose (Bhatt et al., 2008). These labile precursors gave rise to palmitone derivatives following alkali treatment, which were visualised on Thin Layer Chromatography (TLC) plates migrating close to the solvent front in a solvent system used for separating methyl esters of mycolic acids and fatty acids (FAMEs and MAMEs) (Bhatt et al., 2008). To test the effects of conditional depletion of the mycolyl reductase we labelled the cultures of BCG::P_Tet_‐*BCG2529* with ^14^[C]‐acetic acid following grown in the presence and absence of ATc, and extracted MAMEs from the cultures. TLC analysis of the extracted MAMES showed a decrease in the levels of α and keto MAMES and an accumulation of the degradation products derived from 3‐oxo‐mycolate precursors close to the solvent front (Figure 7). These results showed that, as in *M. smegmatis*, loss of the mycolyl reductase function led to the loss of mature mycolic acid production and accumulation of the premature 3‐oxo‐mycolate precursors in slow grow mycobacteria.

## 3 DISCUSSION

As summarised above, the predicted structure of the Rv2509 displays a Rossmann fold with seven parallel β‐strands forming a central beta‐sheet, which is sandwiched between two layers of alpha‐helices—three on each side (Figure 4). While largely consistent with the “classical” family of SDRs (Persson et al., 2003), in *Mycobacteriaceae*, the NADP(H)‐binding motif (G‐x‐x‐Q‐N‐I‐G), alongside the core stabilisation consensus (C‐A‐N‐A‐G‐T) is significantly divergent from the consensus, to instantly identify this group of SDRs, and in our view is distinct enough to be separated into a distinct family.

While we currently lack knowledge about the exact mode of cofactor and ligand binding within the Rv2509 family, some information could be inferred from the structurally related FabG family of β‐ketoacyl reductases which are classified as “Complex family SDRs” (Persson et al., 2003). Given that the structural overlay of Rv2509 models with existing ligand‐bound FabG members yields an RMSD of around 1 Å over the C‐alpha backbone atoms, and that FabG share common active site residues with *Rv2509* it is tempting to suggest that these structures share common ligand‐binding and NADP(H) orientations towards the active site residues with that observed in the aceto‐acyl‐CoAs. Furthermore, the predicted 3D structure of Rv2509 also revealed a unique α‐helical C‐terminal extension, which we subsequently showed to be essential for function—constructs with deletions in the C‐terminal failed to complement (restore mycolate biosynthesis) in the *M. smegmatis* mutant.

The role of this C‐terminal domain is difficult to assign with certainty; however, in related SDRs similar C‐terminal extensions play a number of functions. In the NADP(H)‐dependent tetrameric serine‐dehydrogenase YdfG from *E. coli* (3ASV.pdb) (Yamazawa et al., 2011), the C‐terminal region protrudes from the main core of the protein and is required for tetramer formation, while it also plays a role in the substrate binding *via* stabilisation of the substrate loop. It is also notable that in FabG family of related SDRs discussed above also organise as tetrameric assemblies with the participation of their C‐terminal domains (Javidpour et al., 2014). Furthermore, in the mycobacterial FabG4 dimer, Arg146 and Arg445 of one protomer interact with the C‐terminus of the second protomer and play an essential role in the substrate association and catalysis (Dutta, Bhattacharyya, Roychowdhury, Biswas, & Das, 2013). However, our experiments with purified full‐length and truncated Rv2509 do not seem to suggest that the mycolyl reductase forms oligomers.

While it isn't in itself surprising that Rv2509 presents a conserved SDR fold, our analysis has identified it for the first time decisively as a member of the NADPH‐binding family and indicated that it possesses a number of unique features. Taken together with the unique signatures of the NADPH‐binding site, core stabilisation domains, as well as C‐terminal domain extension, the unique features of Rv2509 possibly merit its inclusion into a distinct family**.**

Our previous studies with *M. smegmatis* suggested that the mycolyl reductase would likely not be essential for the growth in *M. tuberculosis* and related slow‐growing mycobacteria. However, this work demonstrated that gene encoding the reductase, *Rv2509*, was an essential gene required for the growth of slow‐growing mycobacteria in laboratory media. This, along with the identification of unique domains in the predicted structure of Rv2509 highlights its potential as a novel anti‐TB drug target.

## 4 EXPERIMENTAL PROCEDURES

### 4.1 Growth conditions, strains, phages and plasmids

*M. smegmatis* mc^2^155 and derived strains was cultured in Tryptic Soy Broth (TSB) or on TSB‐agar, *M. bovis* BCG strains were grown in 7H9 broth or on 7H10 agar plates. *E. coli* strains were grown in LB broth or LB agar. Hygromycin was used at a concentration of 100 mg/ml for *E. coli* and mycobacterial strains. Kanamycin was used at a concentration of 25 mg/ml and 50 mg/ml for mycobacteria and *E. coli* respectively. Recombinant mycobacterial strains, phages and plasmid constructs used in this study are listed and described in Table 1.

**Table 1:** Plasmids and bacterial strains used in this study

	Description	Reference/Source
Plasmids		
pMV261	*E. coli*‐*Mycobacterium* shuttle plasmid vector with *hsp60* promoter and Kan^R^ cassette (*aph*)	Stover et al. (1991)
pMV261‐*Rv2509*	*Rv2509* cloned in pMV261	Bhatt et al. (2008)
pMV261‐*NCgl2385*	*NCgl2385* cloned in pMV261	This work
pMV261‐*Rv2509D1*	Deletion construct of *Rv2509* cloned in pMV261 encodes a truncated protein missing the C‐terminal residues Ala_255_‐Ser_268_	This work
pMV261‐*Rv2509D2*	Deletion construct of *Rv2509* cloned in pMV261 encodes a truncated protein missing the C‐terminal residues Tyr_262_‐Ser_268_	This work
pMV261::*rev‐tetR*‐RBS‐D	Episomal *E. coli*‐mycobacterium shuttle plasmid pMV261::*rev‐tetR*‐RBS‐D providing constitutive *rev‐tetR* gene expression from the HSP60 promoter in mycobacteria	Korte et al. (2016)
pET28a‐*Rv2509*	Plasmid for expressing *Rv2509* in *E. coli*	This work
pET28a‐*Rv2509D1*	Plasmid for expressing C‐terminal truncated *Rv2509* in *E. coli*	This work
Bacterial strains		
mc^2^155	Electroporation‐proficient *ept* mutant of *M. smegmatis* strain mc^2^6	Snapper, Melton, Mustafa, Kieser, and Jacobs (1990)
Δ*MSMEG4722*	Deletion mutant of mc^2^155 in which *MSMEG4722* is replaced by *hyg*	Bhatt et al. (2008)
Δ*MSMEG4722*‐*CRv*	Δ*MSMEG4722*‐containing pMV261‐*Rv2509*	Bhatt et al. (2008)
Δ*MSMEG4722*‐*CNCgl2385*	Δ*MSMEG4722*‐containing pMV261‐*NCgl2385*	This work
Δ*MSMEG4722‐*CRvD1	Δ*MSMEG4722*‐containing pMV261‐*Rv2509D1*	This work
Δ*MSMEG4722‐*CRvD2	Δ*MSMEG4722*‐containing pMV261‐*Rv2509D2*	This work
BCG::P_Tet_‐*BCG2529*	BCG Pasteur strain containing the P*myc1* promoter from *M. smegmatis* engineered to contain four *tetO* operator sites, inserted immediately upstream of the start codon of *BCG2529*, the BCG homologue of *Rv2509*	This work

### 4.2 Sequence analysis and homology modelling

The multiple sequence alignments (MSA) were prepared using MAFFT and NJ/UPGMA phylogeny algorithms as implemented in MAFFT v.7 server (https://mafft.cbrc.jp/ (Katoh, Misawa, Kuma, & Miyata, 2002)) and the resulting phylogenetic trees were visualised using Archaeopteryx (Han & Zmasek, 2009). Multiple sequence alignment visualisation and structural annotations were performed using ESPript 3 (Gouet, Robert, & Courcelle, 2003). For the purposes of homology modelling, we employed I‐TASSER (Yang et al., 2015) with the assignment of templates. Outputs were independently corroborated by ab‐initio modelling using the New Robetta server (http://new.robetta.org). Sequence conservation analysis was performed using ConSurf (Ashkenazy et al., 2016) and visualised using PyMOL (The PyMOL Molecular Graphics System, Version 1.71 Schrödinger, LLC) with additional local manual refinement and structural superposition performed in Coot (Emsley, Lohkamp, Scott, & Cowtan, 2010). Secondary structure predictions for the C‐terminal domain were performed with JPred4 (Cole et al., 2008) and PSIPRED (Buchan et al., 2013).

### 4.3 Expression, purification and analysis of Rv2509 and its C‐terminal truncated derivative

Rv2509 was cloned in the expression vector pET28a by PCR from *M. tuberculosis* H37Rv genomic DNA using the primer pair Pet28a_Rv2509 _F2 (5′‐TTTACATATGCCGATA CCCGCGCCC‐3′) and Pet28a_Rv2509_R1 (5′‐ATTAAAGCTTCTAGCTGCCCCCAA GCCTCTTG‐3′). Similarly, the C‐terminal truncated version of Rv2509 (missing the last 14 amino acids) was PCR amplified using the primer pair Pet28a_Rv2509 _F2 (5′‐TTTACATA TGCCGATACCCGCGCCC‐3′) and Pet28a_Rv2509_delR1 (5′‐ATTAAAGCTTCTACA CGATGGCGCGCGGAGC‐3′). The constructs were cotransformed into BL21 cells with *M. tuberculosis* chaperone GroES 60.2. Cultures were seeded 1:100 with an overnight starter culture and grown at 37°C in terrific broth supplemented with ampicillin (100 µg/ml) and kanamycin (50 µg/ml) to OD600 = 0.6 and induced with 0.5 mM IPTG overnight at 37°C or 16°C respectively. Cells were harvested at 4,000 rpm at 4°C, washed with PBS and frozen at −80°C until further use. Full‐length protein was purified on cobalt‐IMAC in buffer 50 mM NAH2PO4 pH 7.5, 500 mM NaCl, 10% glycerol and truncated in the same buffer composition at pH 7.0. The eluted fraction of 200 mM imidazole was taken for native‐PAGE analysis.

A native polyacrylamide gel with the separated samples was transferred to a Hybond™ nitrocellulose membrane (GE Healthcare Life Sciences) which was then blocked in 5% milk 20 mM Tris pH 7.5, 150 mM NaCl TBS 0.05% Tween for 1h at room temperature followed by incubation with 0.1% milk TBS‐tween with penta‐his antibody BSA free (Qiagen). Following washing with TBS‐tween the membrane was incubated with 0.1% milk TBS‐tween with anti‐mouse IgG‐Alkaline Phosphatase (Sigma Aldrich) for 30 min at room temperature, washed with TBS‐tween and a final wash with TBS. The membrane was stained for 5 min with BCIP/NBT (SIGMAFAST™) in water for visualisation.

Cross‐linking of full‐length Rv2509 was carried out in 50 mM NaH_2_PO_4_ pH 7.5, 10% glycerol at a final concentration of 0.1% glutaraldehyde and allowed to progress at room temperature for 2 min. The reaction was halted with 1M Tris pH 8 and subsequently run on SDS‐PAGE for visualisation using SYPRO‐ruby (Molecular Probes).

### 4.4 Conditional depletion of the Rv2509 homologue *BCG2529* in *M. bovis* BCG‐Pasteur

For establishing the regulated expression of the *BCG2529* gene, a synthetic gene cassette (*hyg*‐P*myc1*‐4X*tetO*) comprising a hygromycin‐resistance gene and the P*myc1* promoter from *M. smegmatis* engineered to contain four *tetO* operator sites (Korte et al., 2016) was inserted immediately upstream of the *BCG2529* start codon in *M. bovis* BCG‐Pasteur. Targeted gene knock‐in was achieved by specialised transduction employing temperature‐sensitive mycobacteriophages essentially as described previously (Korte et al., 2016). Briefly, for the generation of an allelic exchange construct for site‐specific insertion of the *hyg*‐P*myc1*‐4X*tetO* cassette in *M. bovis* BCG‐Pasteur, upstream‐ and downstream DNA regions flanking the *BCG2529* start codon were amplified by PCR employing the oligonucleotide pairs *Mb2537*_LL 5′‐TTTTTCCATAAATTGGAACCGCTACCTGACATGAAACCC‐3′ and *Mb2537*_LR 5′‐TTTTTCCATTTCTTGGGCCGATGTTCTGCGAAGCCCCGG‐3′ as well as *Mb2537*_RL 5′‐TTTTTCCATAGATTGGATGCCGATACCCGCGCCCAGCCC‐3′ and *Mb2537*_RR 5′‐TTTTTCCATCTTTTGGCGGTGTGGGAGGAGATACTCAAG‐3′ respectively. The gene *BCG2528c* is localised in antilinear orientation upstream of *BCG2529* with only 84 bp between the start codons of both genes. Since it could not be excluded that the *Mb2536c* promoter region might overlap with the *BCG2529* coding region, the upstream flanking region contained a duplication of 66 bp of the 5′‐end of *BCG2529* to conserve 150 bp in front of *BCG2529* that likely comprised its promoter. Subsequently, the upstream and downstream flanks were digested with *Van*91I (restriction sites underlined) and ligated with *Van*91I‐digested pcRv1327c‐4XtetO vector arms (Korte et al., 2016). The resulting knock‐in plasmid was then linearised with *Pac*I and cloned and packaged into the temperature‐sensitive phage phAE159 (Jain et al., 2014), yielding a knock‐in phage which was propagated in *M. smegmatis* at 30°C. Allelic exchange in *M. bovis* BCG‐Pasteur using the knock‐in phage at the nonpermissive temperature of 37°C was achieved by specialised transduction using hygromycin (50 mg/L) for selection, resulting in the site‐specific insertion of the *hyg*‐P*myc1*‐4X*tetO* cassette. The obtained *M. bovis* c‐BCG_2529‐4XtetO knock‐in mutant was verified by the diagnostic PCR of genomic DNA using the oligonucleotide pair BCG_2529_L_fw 5′ GTCAGGTAGACGGAGAACAC‐3′ and BCG_2529_L_rev 5′ AGCTCACCGCGCAGAGATTC‐3′ binding outside the allelic exchange substrates used to generate this mutant and subsequent sequencing of PCR products (Supporting information Figure S6). For achieving the controlled gene expression of the *Mb2537* gene, a synthetic gene (*rev‐tetR*) derived from Tn10 *tetR* encoding a mutated TetR protein with reversed binding affinity to *tetO* sites upon the binding of tetracycline (Klotzsche, Ehrt, & Schnappinger, 2009) was heterologously expressed in the knock‐in mutant by electroporation of the episomal *E. coli*‐mycobacterium shuttle plasmid pMV261::*rev‐tetR*‐RBS‐D providing constitutive *rev‐tetR* gene expression from the HSP60 promoter in mycobacteria using solid medium containing 50 mg/L of hygromycin and 20 mg/L of kanamycin for selection (Famulla et al., 2016). This yielded the conditional mutant BCG‐Pasteur c‐*BCG2529*‐4X*tetO* pMV261::*rev‐tetR*‐RBS‐D (referred to as BCG::P_Tet_‐*BCG2529*) allowing silencing of the *BCG2529* gene in the presence of anhydrotetracycline (ATc).

For silencing experiments, precultures of the BCG::P_Tet_‐*BCG2529* strain were first grown in liquid medium containing 0.3 µg/ml of ATc, before these precultures were used to inoculate the test cultures containing 0–5 µg/ml of ATc. Growth in 96‐well microtitre plates was quantified after incubation at 37°C for five days by adding 10% of 100 µg/ml of resazurin solution. After further incubation at ambient temperature for 16 hr, cells were fixed for 30 min by formalin addition (5%, v/v, final concentration) and fluorescence was quantified using a microplate reader (excitation 540 nm, emission 590 nm). For the growth on 7H10 agar, nonpermissive conditions were obtained by adding ATc to a final concentration of 5 µg/ml.

### 4.5 Functional complementation studies

Deletion constructs for *Rv2509* were generated by PCR amplification of selected regions of *Rv2509* using pMV261‐*Rv2509* as a template, incorporating a premature stop codon in the reverse primer. The plasmid constructs generated in this manner were called pMV261‐Rv2509D1 and pMV261‐Rv2509D2, encoding a truncated protein missing the C‐terminal residues Ala_255_‐Ser_268_ and Tyr_262_‐Ser_268_ respectively. The *C. glutamicum* *Rv2509* orthologue, *NCgl2385* was PCR amplified using *C. glutamicum* genomic DNA as a template and the PCR product was cloned into pMV261 using primer incorporated BamHI and EcoRI sites generating the plasmid pMV261‐*NCgl2385*. Plasmid clones were electroporated into the *M. smegmatis* Δ*MSMEG4722* mutant strain using previously described protocols (Snapper et al., 1990). Kanamycin‐resistant transformants were cultured in TSB and FAMEs and MAMEs were extracted from the cell pellets and separated by TLC as previously described (Vilcheze & Jacobs, 2007).

### 4.6 RT‐PCR of *Rv2509* transcripts

*M. smegmatis* strains were grown to mid‐log phase and RNA was extracted by acid phenol and chloroform extraction. The purified RNA was treated with DNase and converted to cDNA, which was used as a template for quantitative PCR. PCR products were run on a 1% agarose gel to detect the expression of *Rv2509*. The gene *sigA* was monitored as a constitutively expressed gene. The primers used for PCR amplification were Rv2509RT‐f (5′‐ACAAGTACCGCGTCACGGTC ‐3′) and Rv2509RT‐r (5′‐AAGTCCGGCACC AGCTTCTC‐3′) for the amplification of nondeleted sections of *Rv2509*.

## AUTHOR CONTRIBUTIONS

AB, RK and VB designed the study. AJ, CC, AS, SS, MH and RM conducted experiments and acquired data. AJ, CC, AS, SS, MH, RM, AB, RK and VB interpreted the data. AB, RK and VB wrote the manuscript.

## Supporting information

FigS1‐S6
