# Supplementary material for: The mycolic acid reductase Rv2509 has distinct structural motifs and is essential for growth in slow‐growing mycobacteria
Source: Mol Microbiol. 2019 Dec 17;113(2):521–33. doi: 10.1111/mmi.14437 (PMC7065075; doi:10.1111/mmi.14437)
Supplement: Supplementary file 1 — FigS1‐S6 [file MMI-113-521-s001.pptx]

## Slide 1
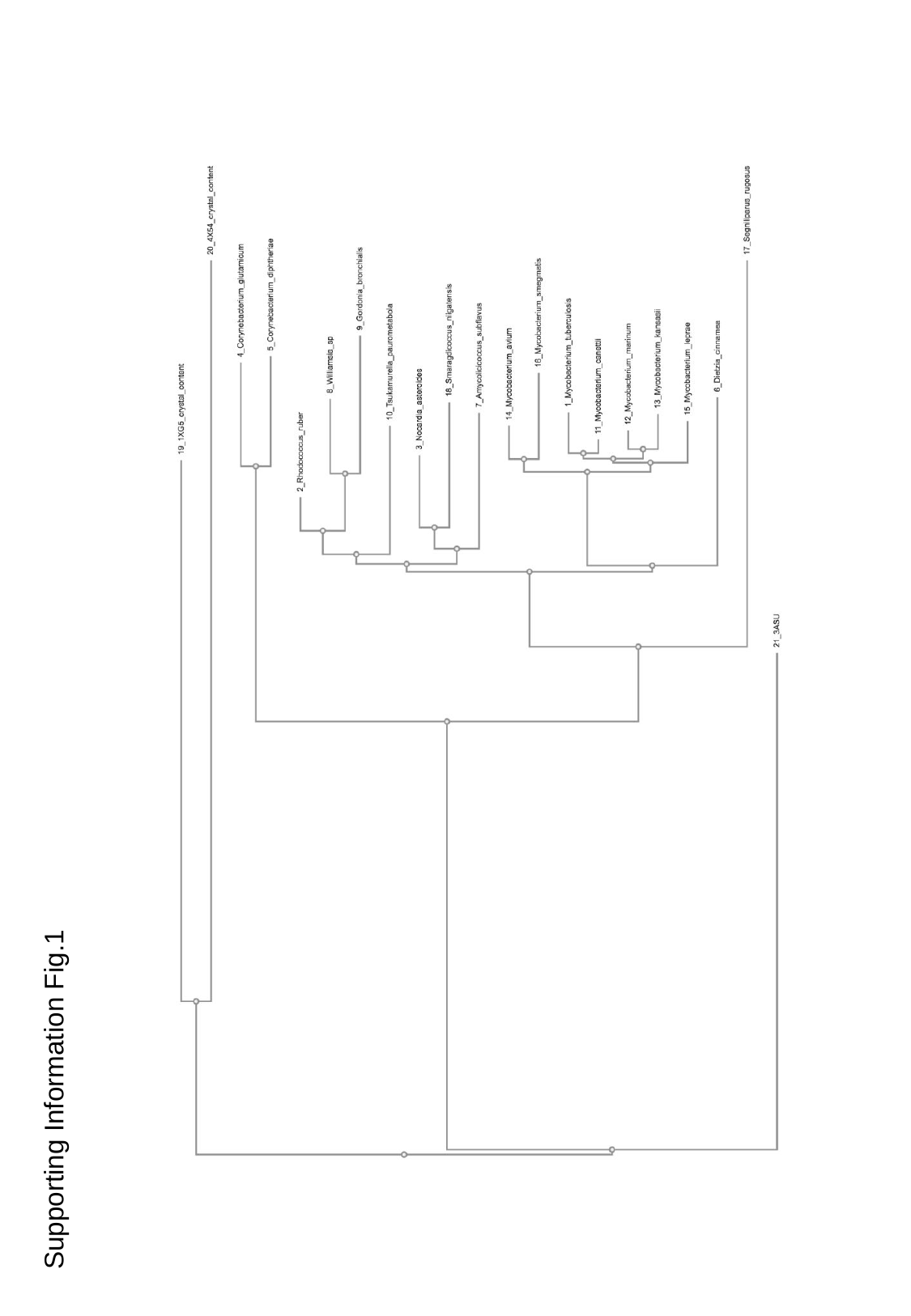

Supporting Information Fig.1

## Slide 2
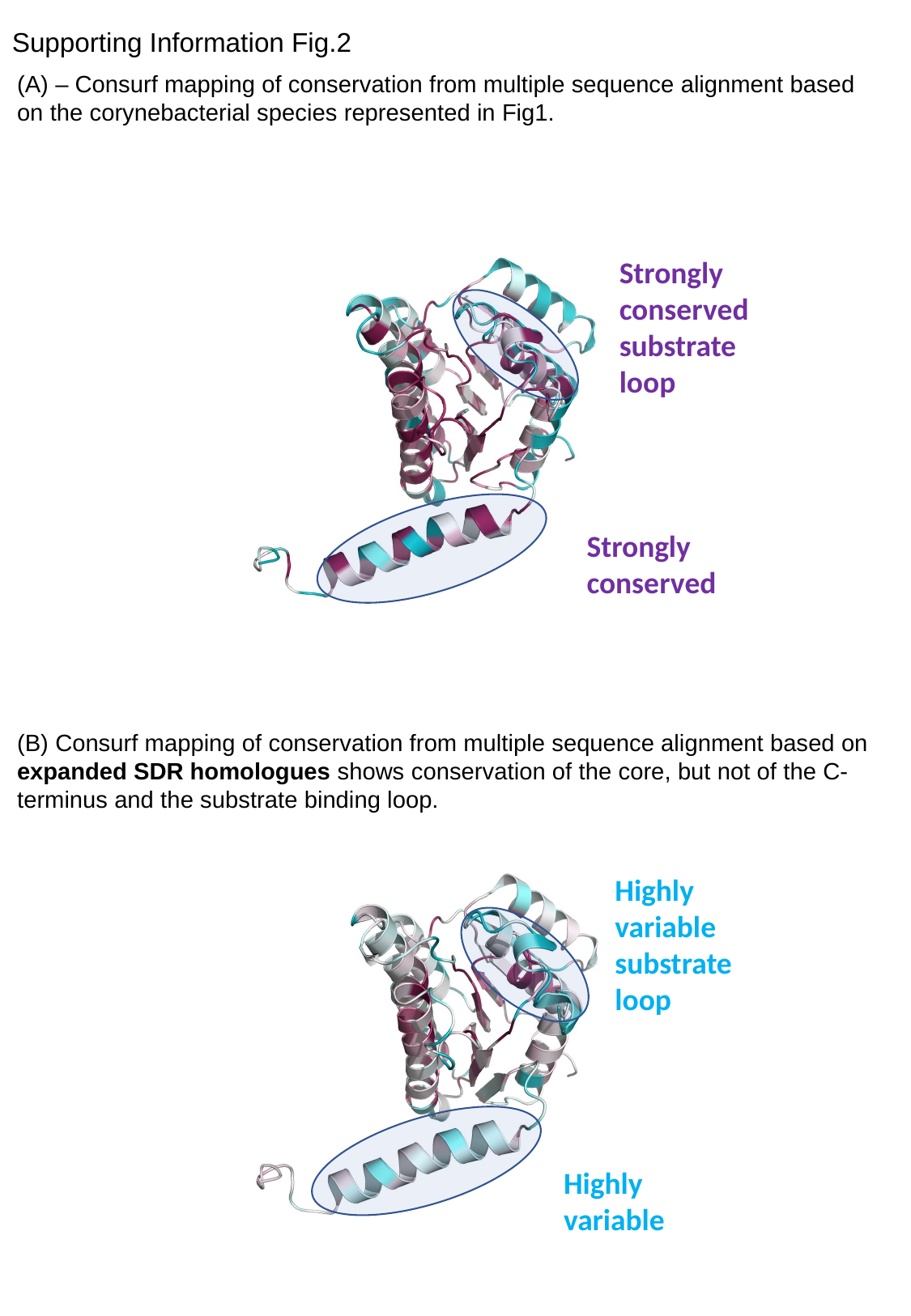

Supporting Information Fig.2
(A) – Consurf mapping of conservation from multiple sequence alignment based on the corynebacterial species represented in Fig1.
Strongly conserved
substrate loop
Strongly conserved
(B) Consurf mapping of conservation from multiple sequence alignment based on expanded SDR homologues shows conservation of the core, but not of the C-terminus and the substrate binding loop.
Highly variable
substrate loop
Highly variable

## Slide 3
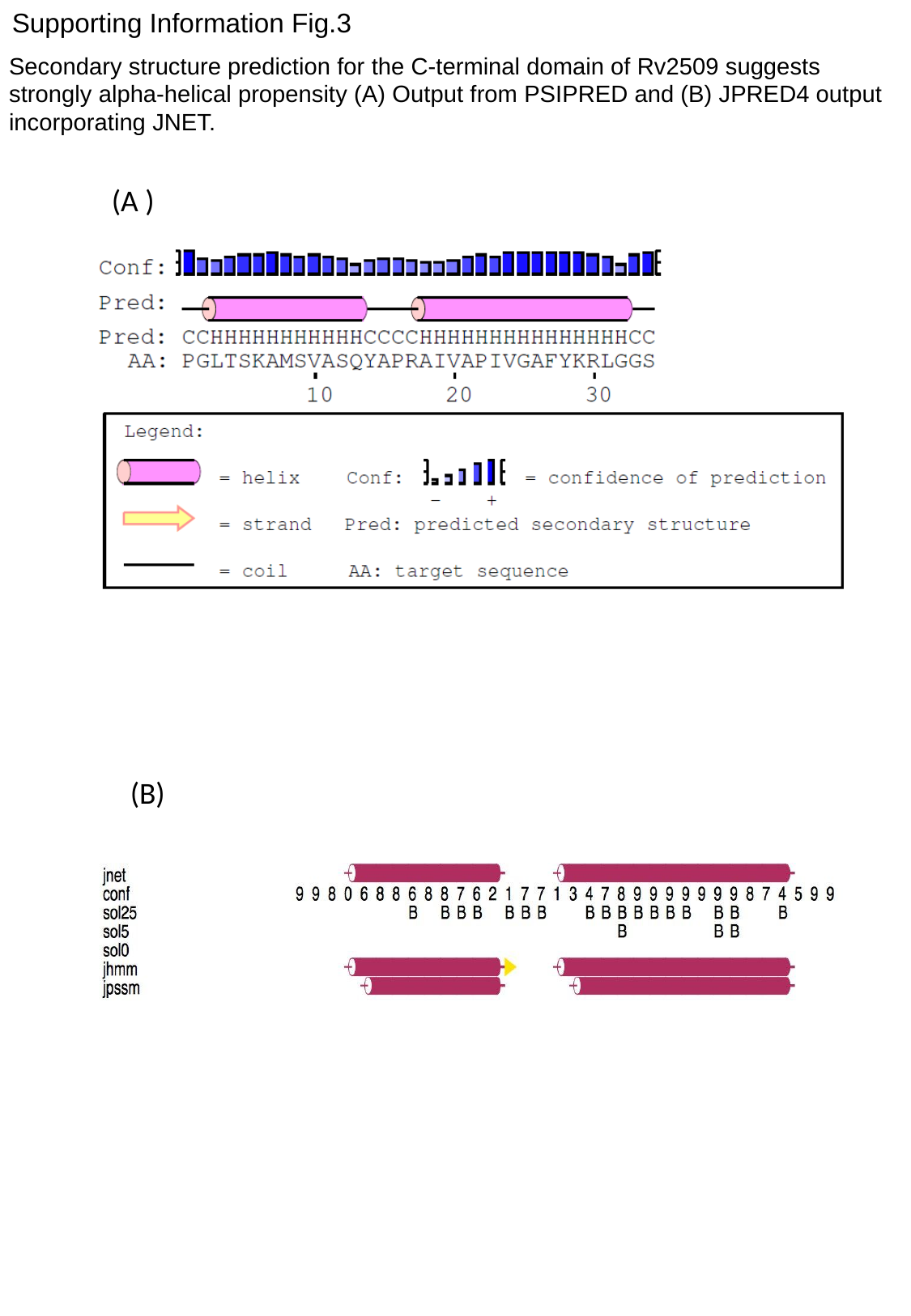

Supporting Information Fig.3
Secondary structure prediction for the C-terminal domain of Rv2509 suggests strongly alpha-helical propensity (A) Output from PSIPRED and (B) JPRED4 output incorporating JNET.
(A )
(B)

## Slide 4
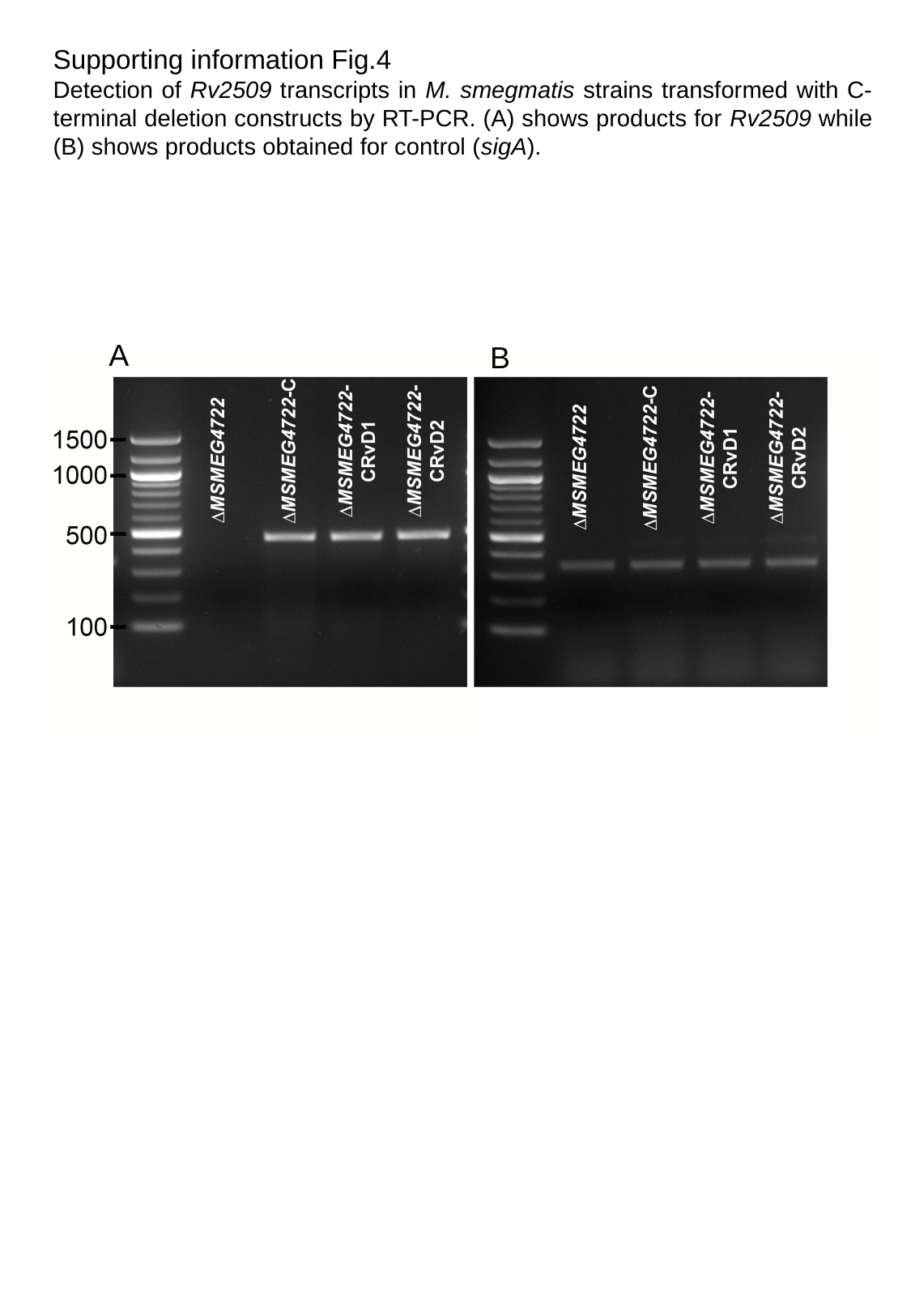

Supporting information Fig.4
Detection of Rv2509 transcripts in M. smegmatis strains transformed with C-terminal deletion constructs by RT-PCR. (A) shows products for Rv2509 while (B) shows products obtained for control (sigA).
A
B

## Slide 5
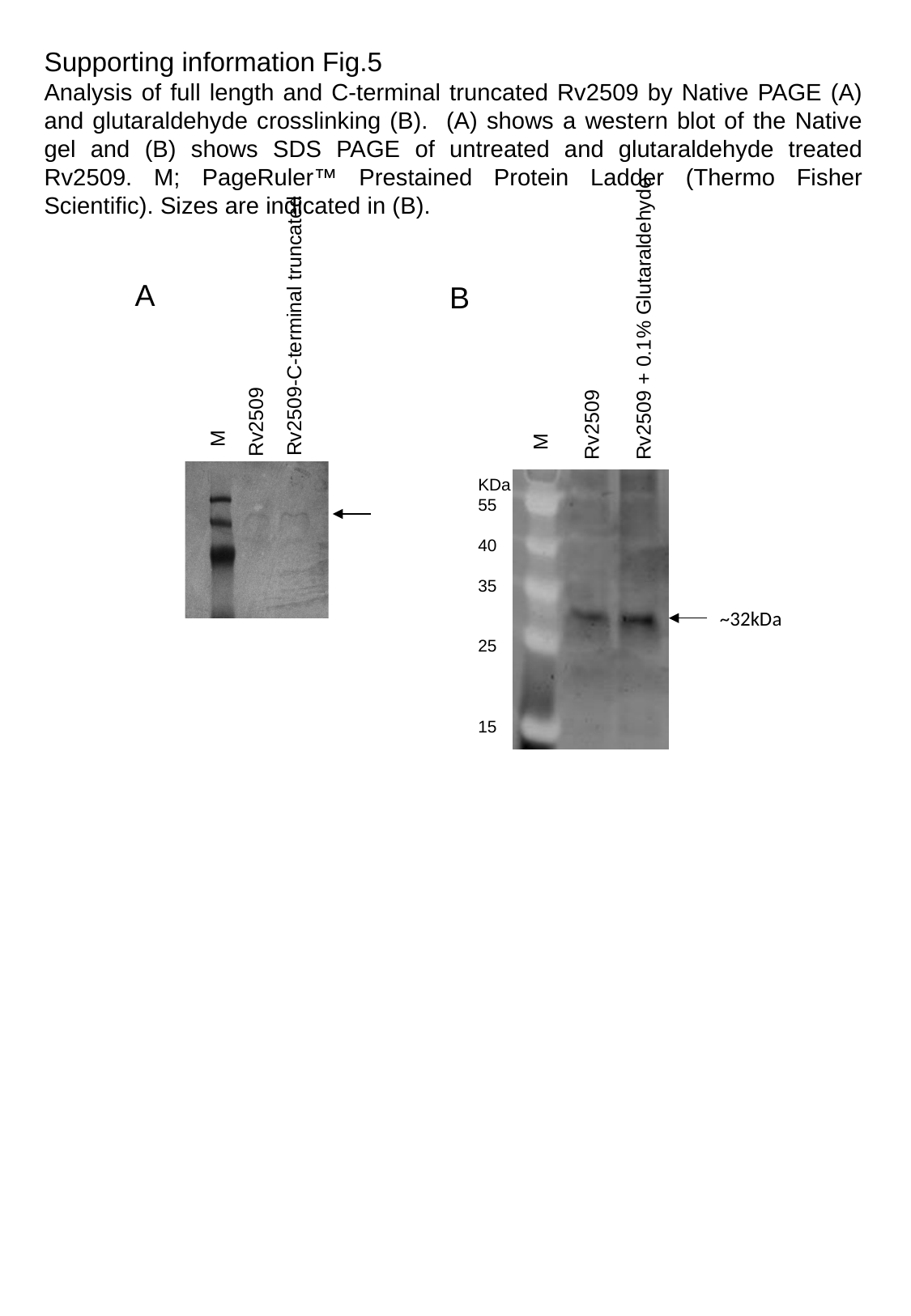

Supporting information Fig.5
Analysis of full length and C-terminal truncated Rv2509 by Native PAGE (A) and glutaraldehyde crosslinking (B). (A) shows a western blot of the Native gel and (B) shows SDS PAGE of untreated and glutaraldehyde treated Rv2509. M; PageRuler™ Prestained Protein Ladder (Thermo Fisher Scientific). Sizes are indicated in (B).
A
B
Rv2509 + 0.1% Glutaraldehyde
Rv2509-C-terminal truncated
Rv2509
Rv2509
M
M
KDa
55
40
35
25
15
~32kDa

## Slide 6
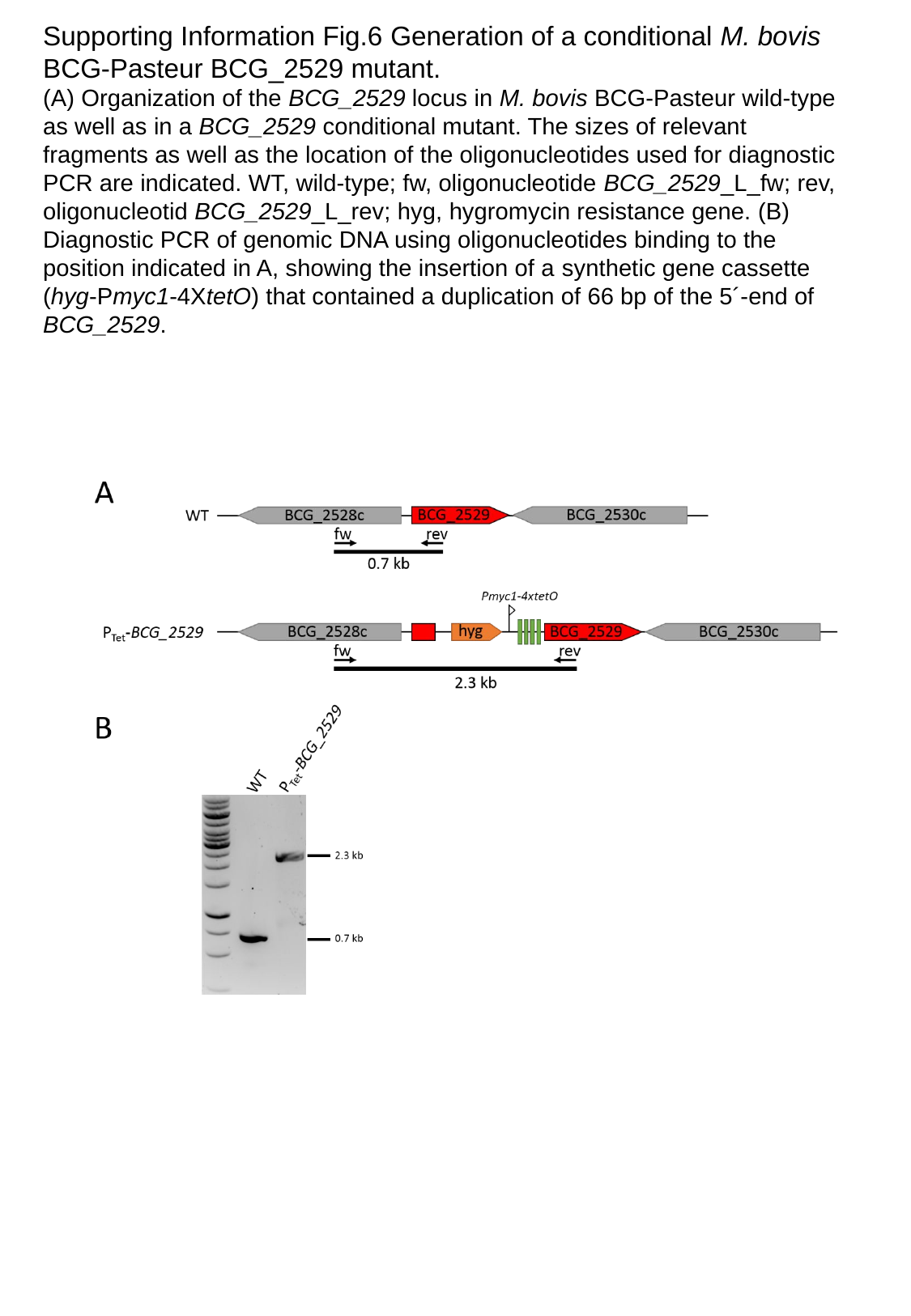

Supporting Information Fig.6 Generation of a conditional M. bovis BCG-Pasteur BCG_2529 mutant.
(A) Organization of the BCG_2529 locus in M. bovis BCG-Pasteur wild-type as well as in a BCG_2529 conditional mutant. The sizes of relevant fragments as well as the location of the oligonucleotides used for diagnostic PCR are indicated. WT, wild-type; fw, oligonucleotide BCG_2529_L_fw; rev, oligonucleotid BCG_2529_L_rev; hyg, hygromycin resistance gene. (B) Diagnostic PCR of genomic DNA using oligonucleotides binding to the position indicated in A, showing the insertion of a synthetic gene cassette (hyg-Pmyc1-4XtetO) that contained a duplication of 66 bp of the 5´-end of BCG_2529.
